# Advancements in the non-invasive diagnosis of renal fibrosis

**DOI:** 10.3389/fmed.2025.1646412

**Published:** 2025-07-30

**Authors:** Tingting Yuan, Hong Wang, Ting Kang, Weihua Wu, Santao Ou

**Affiliations:** ^1^Department of Nephrology, The Affiliated Hospital of Southwest Medical University, Luzhou, China; ^2^Sichuan Clinical Research Center for Nephrology, Luzhou, China; ^3^Metabolic Vascular Disease Key Laboratory of Sichuan Province, Luzhou, China

**Keywords:** chronic kidney disease, renal fibrosis, non-invasive diagnosis, biomarkers, imaging techniques

## Abstract

Renal fibrosis is the central pathological pathway by which various primary and secondary kidney diseases progress to end-stage renal disease. It is characterized by excessive extracellular matrix deposition and destruction of the native renal parenchyma, ultimately leading to irreversible loss of nephrons. Currently, percutaneous renal biopsy with histopathological evaluation remains the diagnostic gold standard for renal fibrosis, allowing semiquantitative scoring of renal interstitial fibrosis and glomerulosclerosis (e.g., Banff grading). However, this invasive procedure carries a risk of bleeding and is limited by sampling error and inter-observer variability, making it impractical for dynamic disease monitoring. In recent years, significant advances have been made in noninvasive diagnostic techniques. These include: (1) blood and urine biomarkers such as markers of ECM metabolism, inflammatory factors, tubular injury markers, and extracellular vesicles; (2) imaging modalities including novel ultrasound techniques, shear wave elastography, functional magnetic resonance imaging (MRI) methods such as diffusion-weighted imaging, blood oxygen level-dependent MRI, magnetic resonance elastography, and positron emission tomography/computed tomography using radiotracers targeting fibrosis-associated molecules such as ^68^Ga-FAPI. This review systematically summarizes the latest evidence on the above biomarkers and advanced imaging modalities, with an emphasis on their diagnostic performance (sensitivity/specificity), utility for dynamic monitoring, and bottlenecks in clinical translation. The aim is to develop a multimodal, noninvasive assessment system to enable earlier fibrosis detection, stratified disease management, and precise intervention targeting fibrogenic pathways, ultimately improving renal disease outcomes.

## 1 Introduction

Chronic kidney disease (CKD) is defined as structural or functional kidney damage, manifesting as an estimated glomerular filtration rate (eGFR) below 60 mL/min/1.73 m^2^ or urinary protein ≥30 mg/day for over 3 months (1, 2), in 2017, approximately 843.6 million people worldwide were affected by CKD. Although the mortality of patients with end-stage renal disease has declined, the Global Burden of Disease study showed that CKD has become one of the leading global causes of death (3).

**Figure 1:**
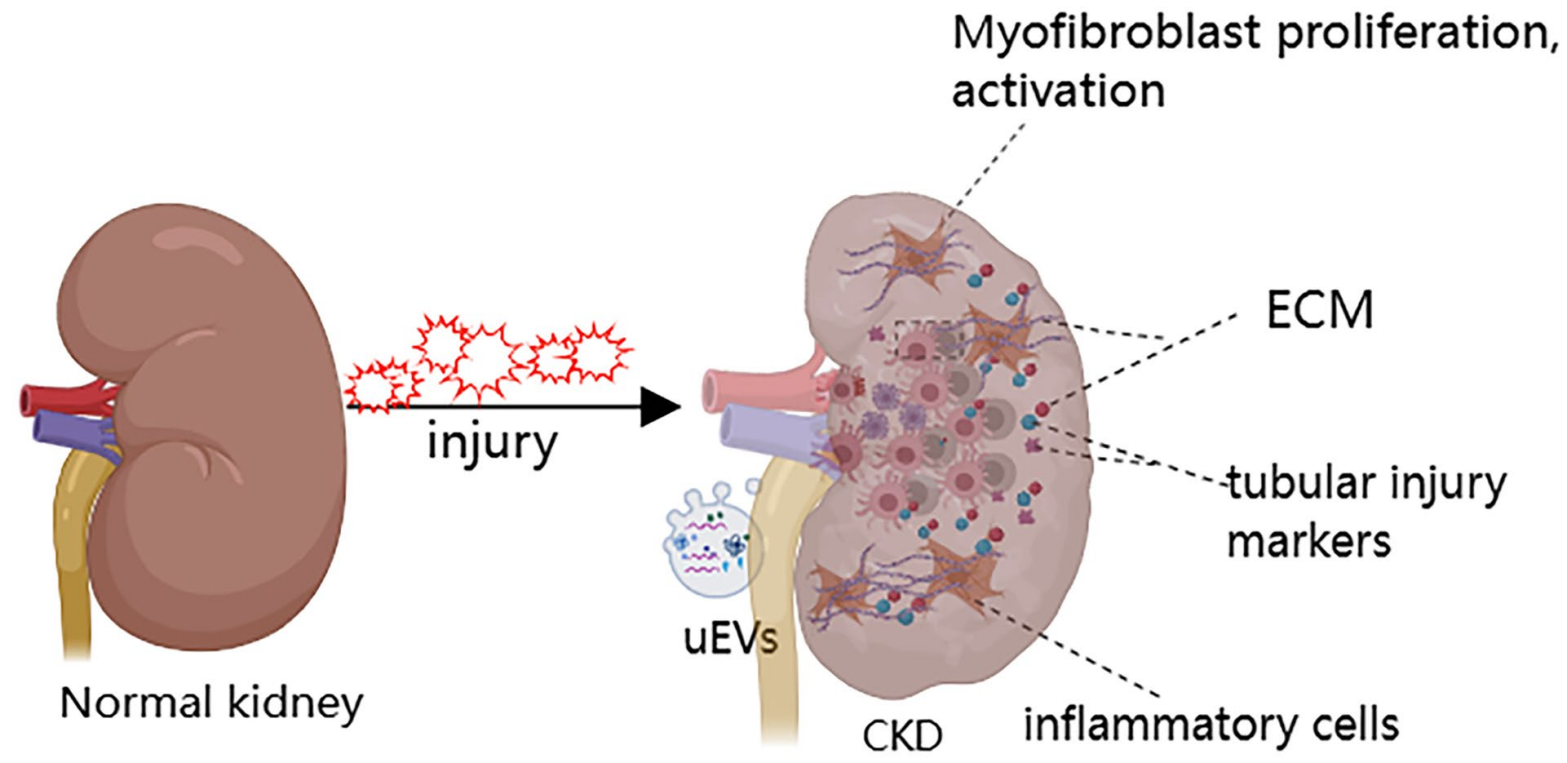
Renal fibrosis is the final common pathophysiological pathway of CKD.

**Figure 2:**
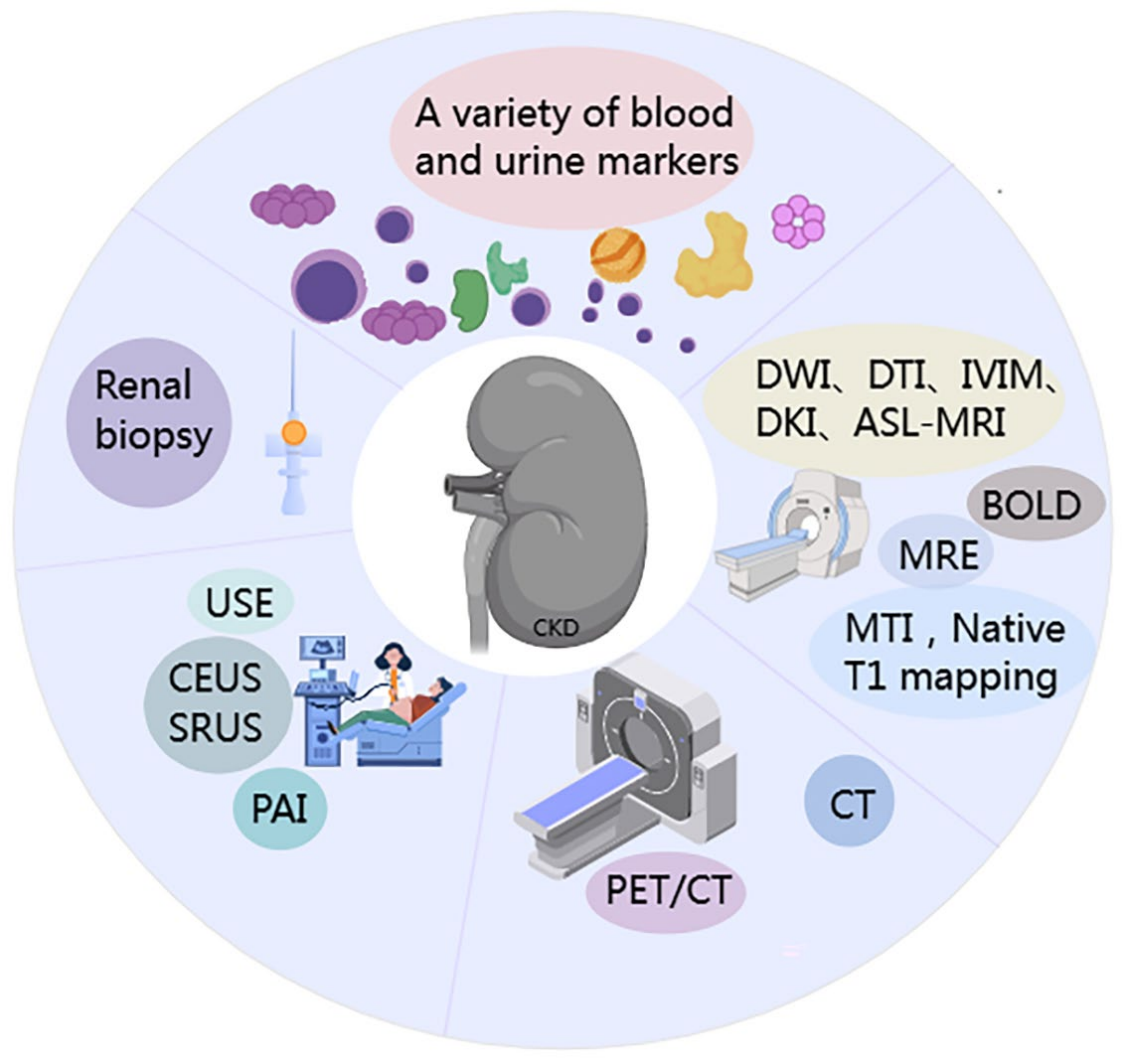
Schematic of methodological approaches for renal fibrosis (RF) assessment.

Renal fibrosis (RF) is characterized by excessive extracellular matrix (ECM) deposition leading to scar formation, representing the common outcome of various CKD (4–6) (Figure 1). Currently, clinical evaluation of CKD relies mainly on eGFR and proteinuria. However, these indicators have significant limitations: due to the kidney’s functional reserve, eGFR cannot detect early renal fibrosis and cannot assess the extent of interstitial fibrosis (7). In addition, kidney biopsy is the diagnostic gold standard for renal fibrosis, but its clinical use faces many challenges: it is invasive, allows only limited tissue sampling, has low repeatability, and provides limited accuracy in grading fibrosis severity. In recent years, blood and urine biomarker assays combined with advanced imaging techniques-such as ultrasound and magnetic resonance imaging (MRI), and especially positron emission tomography/computed tomography (PET/CT) (Figure 2) have emerged as important noninvasive means to evaluate renal fibrosis. These technologies offer the promise of early and accurate fibrosis detection, and they support dynamic monitoring of disease progression and timely interventions to slow CKD progression.

We reviewed the literature on blood/urine biomarkers and imaging modalities for the noninvasive diagnosis of renal fibrosis in the past 5 years, to evaluate CKD progression and fibrosis severity in comparison with traditional indicators like eGFR and proteinuria, with a focus on clinical applicability.

## 2 Blood and urine biomarkers of renal fibrosis

Persistent tubular injury, inflammatory activation, and collagen/ECM deposition are considered major drivers of renal fibrosis. Based on these key pathological events, an increasing number of serum and urine biomarkers have been identified, providing new avenues for the clinical assessment of renal fibrosis.

### 2.1 Extracellular matrix

#### 2.1.1 Procollagen type III N-terminal propeptide and procollagen type VI N-terminal propeptide; C1M and C3M

Procollagen type III N-terminal propeptide (PRO-C3) and procollagen type VI N-terminal propeptide (PRO-C6) are biomarkers reflecting the formation of type III and VI collagen, whereas C1M and C3M are fragments generated by matrix metalloproteinase (MMP) degradation of type I and III collagen during ECM remodelling. Studies have found, for example, that ELISA measurement of urinary DKK-3, PRO-C6, and C3M levels in patients with ANCA-associated vasculitis (AAV) versus healthy controls showed that uPRO-C6, uC3M, and uDKK-3 were elevated in AAV patients, and uPRO-C6 and uDKK-3 levels were significantly correlated with the degree of renal fibrosis (8); in patients with lupus nephritis (LN), serum and urine PRO-C3 and PRO-C6 are significantly elevated and associate with interstitial fibrosis and tubular atrophy (9). Among IgA nephropathy (IgAN) patients, sPRO-C3 and sC3M correlate with fibrosis extent on biopsy, while urinary C3M/creatinine is inversely correlated with fibrosis. Another study found that uC3M levels decline with increasing CKD stage and are independently and negatively associated with 12-month and 30-month CKD progression and development of end-stage renal disease (10). However, a separate prospective observational study reported that in type 2 diabetic (T2DM) patients with microalbuminuria, higher sC3M was a risk factor for CKD progression and was associated with inflammatory markers (11). Therefore, molecules related to collagen synthesis and degradation are potential biomarkers of renal fibrosis (Table 1). Notably, some studies indicate that blood biomarkers lack specificity, whereas urinary C3M and C1M may have greater diagnostic and prognostic value (12).

**Table 1:** The key biomarkers reflecting the synthesis and degradation of ECM during the process of renal fibrosis.

Marker	Secretory cells/mechanism	Type of molecule	Function association
PRO-C3	Fibroblast; myofibroblast	Protein fragment	Metabolites of type III collagen precursors
PRO-C6	Mesangial cells; fibroblasts	Protein fragment	VI type collagen synthesis marker
C1M	MMPs degrade type I collagen	Polypeptide	Degradation marker of type I collagen
C3M	MMPs degrade type III collagen	Polypeptide	Type III collagen degradation marker

#### 2.1.2 Matrix metalloproteinases

Matrix metalloproteinases (MMPs) are secreted by glomerular cells, renal tubular epithelial cells, and macrophages to degrade the ECM, while TIMPs (tissue inhibitors of metalloproteinases) specifically inhibit MMPs by forming complexes, thus regulating ECM degradation and remodelling (13). Enoksen et al. (14) measured baseline serum levels of eGFR, MMP-2, MMP-7 and TIMP-1 in 1,627 subjects without diabetes, kidney, or cardiovascular disease and re-evaluated them after a median of 5.6 years; they found that the profibrotic biomarker sMMP-7 was associated with accelerated GFR decline and increased risk of incident CKD in middle-aged individuals from the general population. Similarly, in a cohort of 1,181 T2DM patients followed over time, the risk of renal function decline increased with higher baseline sMMP-7 levels (15), in hypertensive patients, those with CKD had significantly elevated urinary MMP-7 (16). Multiple studies have demonstrated that CKD patients, including those with diabetic kidney disease (DKD) and IgAN, have significantly higher MMP-7 levels in blood and urine compared to healthy individuals, and that urinary MMP-7 is an independent predictor of IgAN progression and correlates with RF (17, 18). These findings suggest that elevated MMP-7 in blood or urine is associated not only with current renal function but also with future decline, and that increases in MMP-7 could serve as a potential early noninvasive indicator of CKD development in the context of hypertension and diabetes, enabling earlier therapeutic intervention.

### 2.2 Biomarkers associated with inflammatory activation

#### 2.2.1 Transforming growth factor-β1

Transforming growth factor-β1 (TGF-β1) is a growth factor secreted by many cell types, including inflammatory cells, tubular epithelial cells, and fibroblasts (19). TGF-β1 is considered the most potent profibrotic cytokine and a central mediator of RF, involved in fibroblast transdifferentiation and activation (20, 21). In patients with IgAN, serum TGF-β1 levels are elevated and associate with lower eGFR and higher tubular atrophy/interstitial fibrosis (T) scores on biopsy (22). DKD leads 40% of patients who are diabetic and is the leading cause of CKD worldwide (23, 24). Likewise, patients with DKD show significantly increased TGF-β1 levels (25). Furthermore, in LN patients, urinary TGF-β1 correlates positively with the degree of tubulointerstitial fibrosis (12), suggesting that TGF-β1 in blood or urine may serve as a noninvasive biomarker of renal fibrosis. However, since TGFβ1 broadly participates in fibrosis of other organs and lacks kidney-specificity, further large-scale clinical validation is necessary.

#### 2.2.2 Monocyte chemoattractant protein-1

Monocyte chemoattractant protein-1 (MCP-1), also known as C-C motif chemokine ligand 2 (CCL2), is a chemokine produced by injured tubular epithelial cells and monocytes/macrophages. By inducing inflammatory cell activation and recruiting monocytes/macrophages, MCP-1 mediates and promotes RF (26). Research shows that the measurement of MCP-1 in the urine of DKD patients is higher compared to healthy controls, and it is significantly associated with the progression of CKD, as well as related to changes in urinary albumin levels and eGFR, indicating that uMCP-1 is an important biomarker for assessing the progression of DKD (27, 28). In the SPRINT trial of 2,253 CKD patients, Miller et al. (29) found that uMCP-1 was a marker of tubulointerstitial fibrosis. Research in LN patients likewise showed that uMCP-1 levels are significantly elevated, with biopsy samples revealing marked RF (30). Thus, although MCP-1 is an inflammatory marker, its significant increase at the initiation stage of fibrosis and its correlation with TIF in CKD suggests it has potential utility as a fibrosis biomarker.

#### 2.2.3 Interleukins

Interleukins (ILs) are a group of cytokine proteins produced by various cells (including immune cells) in the body. They can be classified as pro-inflammatory, anti-inflammatory, or dual-effect cytokines based on their biological roles in inflammation (31).

Single-cell RNA sequencing of kidney tissues from CKD patients versus healthy controls found that IL-6, IL-18, and IL-33 expression levels positively correlated with fibrosis severity and negatively with eGFR (32). In patients with DKD, plasma IL-6 concentrations were significantly higher than in controls and were associated with higher proteinuria (33). A cross-sectional study of renal transplant recipients showed that urinary IL-8 was elevated in patients with rejection; thus, urinary IL-8 mRNA may be used as a diagnostic tool for fibrosis (34).

Deng et al. (35) measured plasma IL-7 in IgAN patients and controls, finding that IgAN patients had significantly lower IL-7 levels; IL-7 levels differed between presentation and follow-up, suggesting that sIL-7 may be a noninvasive biomarker for predicting IgAN.

In summary, the interleukin family plays a key role in both promoting and alleviating fibrosis. Interleukins may be potential therapeutic targets and biomarkers, but factors such as IL-6 increase in autoimmune diseases and infections, showing low specificity and making it difficult to distinguish RF from other inflammations. Therefore, there is insufficient evidence for using inflammatory factors in the clinical diagnosis of renal fibrosis.

### 2.3 Tubular injury biomarkers

#### 2.3.1 Kidney injury molecule-1

Kidney injury molecule-1 (KIM-1) is a transmembrane glycoprotein that is highly expressed in injured proximal tubular epithelial cells and can be detected in plasma and urine (36, 37). Studies show that KIM-1 expression in renal tubules correlates with kidney inflammation and fibrosis, and it is considered an early, sensitive, and specific urinary biomarker of kidney injury. KIM-1 can also be used to quantify the severity of tubular cell injury (28, 38). Birnlland et al. (39) evaluated KIM-1 levels in patients with ANCA-associated vasculitis and glomerulonephritis (ANCA-GN) at diagnosis and after treatment. They found that KIM-1 was elevated at diagnosis but decreased after induction of remission therapy, suggesting that KIM-1 may be a biomarker of acute kidney injury (AKI) and tubulointerstitial damage in ANCA-GN. These findings indicate that persistently elevated KIM-1 could serve as an indicator of ongoing renal fibrosis.

#### 2.3.2 Neutrophil gelatinase-associated lipocalin

Neutrophil gelatinase-associated lipocalin (NGAL), also known as lipocalin-2 (LCN-2), is a 25 kDa protein of the lipocalin family. During kidney injury, NGAL is specifically released into the bloodstream and urine (40). In a population-based cohort, higher plasma NGAL concentrations were associated with an increased risk of developing CKD, indicating the potential utility of NGAL as a biomarker for incident CKD risk (41). Research findings indicate that urinary NGAL levels are elevated in T2DM patients compared to healthy controls, and are associated with urine protein levels (36). Using ELISA to quantify urinary NGAL, patients with chronic tubulointerstitial nephritis (D-CTIN), primary membranous nephropathy (PMN), and membranoproliferative glomerulonephritis (MPGN) all showed significantly higher uNGAL levels than healthy controls, and uNGAL was directly proportional to the degree of proteinuria and inversely proportional to residual renal function (42) (Table 1).

#### 2.3.3 N-acetyl-β-D-glucosaminidase

N-acetyl-β-D-glucosaminidase (NAG) is a lysosomal enzyme predominantly present in proximal tubular cells of the kidney; it is not filtered by the glomerulus. Increased urinary NAG excretion is caused entirely by proximal tubular cell injury (43). One study found that uNAG levels were higher in DN patients than in T2DM patients, suggesting that urinary NAG may be an early indicator of disease progression from T2DM to DN (44), KIM-1, MCP-1, and NAG have been identified as the most promising urinary biomarkers for early diagnosis of renal involvement in IgA vasculitis (45).

Nevertheless, Hsu et al. (46) conducted a prospective cohort study and found that after adjusting for known CKD progression risk factors including eGFR and ACR, tubular injury biomarkers such as KIM-1, NGAL, and NAG did not improve prediction of CKD progression. These markers can also be influenced by non-fibrotic conditions like infections; hence, their role as specific indicators of renal fibrosis remains controversial.

#### 2.3.4 Dickkopf-related protein 3

Dickkopf-related protein 3 (DKK-3) is a secreted glycoprotein synthesized by renal tubular epithelial cells under stress conditions (47). ELISA-based measurement of DKK-3 in serum and urine of CKD patients revealed that urinary DKK-3 (uDKK-3) levels were closely correlated with the severity of tubular atrophy (TA) and interstitial fibrosis (IF) observed on kidney biopsy (48). Urinary DKK-3 levels are significantly elevated in renal transplant recipients compared to healthy controls (49). Moreover, uDKK-3 levels increase progressively with advancing CKD stage and correlate inversely with eGFR (47, 50). Elevated DKK-3 also helps identify patients on peritoneal dialysis who are at risk of faster decline in residual renal function (51). Therefore, uDKK-3 has great potential as a biomarker for monitoring renal disease progression, large-scale cohort validation remains necessary.

#### 2.3.5 Vascular cell adhesion molecule 1

Vascular cell adhesion molecule 1 (VCAM1), mainly expressed by activated endothelial cells, shows minimal expression in normal tissue but is highly expressed in fibrotic tissue and involved in cell adhesion (52). Single-cell transcriptomic and LC-MS proteomic analyses revealed significantly elevated VCAM-1 expression in CKD tissues, with higher levels in proliferative LN (PLN) compared to membranous LN (MLN) (53, 54). Serum VCAM-1 measured by ELISA showed significant elevation in CKD patients, correlating with CKD risk in T2DM patients, suggesting its potential for CKD risk stratification in this population (55). Similarly, elevated serum VCAM-1 levels effectively distinguished active LN from healthy controls, remission-phase LN, active non-renal SLE, and non-lupus CKD, correlating positively with proteinuria, Scr, anti-dsDNA antibodies, and negatively with complement C3. Thus, serum VCAM-1 may aid in early detection of LN flares (56). Additionally, integrated GEO database analysis using ML identified VCAM1 as a promising biomarker for renal fibrosis in tissues and serum (57). However, its elevation in conditions such as atherosclerosis and infection reduces kidney-specificity.

### 2.4 Biomarkers in the urine

#### 2.4.1 Extracellular vesicles

Extracellular vesicles (EVs) are membrane-bound vesicles released by cells, mainly originating from renal and other urinary tract cells; they include exosomes, microvesicles, and apoptotic bodies. These vesicles contain proteins, lipids, DNA, mRNA, and microRNAs (miRNAs), reflecting the physiological state of the source cells. EVs thus have potential as novel diagnostic biomarkers (58). Research indicates that urinary release of podocyte-derived exosomal CD2AP mRNA is negatively correlated with the extent of renal fibrosis and glomerulosclerosis, suggesting that CD2AP mRNA could serve as a noninvasive tool to detect renal fibrosis (59). Proteomic analysis of urinary EVs from kidney transplant recipients identified urinary vitronectin (VTN) as a potential independent biomarker for monitoring fibrotic changes in allograft kidneys (60).

miRNAs are small non-coding single-stranded RNAs that regulate gene expression by mRNA degradation or translational inhibition. Cao et al. (61) compared the expression of hsa_circ_0036649 in exosomes from fibrotic versus non-fibrotic patients and found it was correlated with the tubulointerstitial fibrosis (TIF) score and glomerulosclerosis score. Urinary exosomal miRNAs (including miR-21, miR-29, miR-146, and miR-200) may serve as potential biomarkers for early detection of renal fibrosis (62). Zhang et al. (63) measured miR-451a in 40 IgAN patients and found it significantly upregulated, distinguishing patients with mild versus severe tubular atrophy/interstitial fibrosis. Although EVs originate from renal and urinary tract cells and offer advantages in diagnosing and treating renal fibrosis, the lack of standardization in urine collection, processing, and storage for EV analysis, as well as the high inter-individual variability in EV profiles, has hindered the discovery of reliable biomarker candidates (64).

#### 2.4.2 Urine sediment and urinary exfoliated cell

Increasing evidence supports non-invasive urine sediment and urinary exfoliated cell detection for early CKD diagnosis and prognosis (65). Urine sediment examination in DKD patients revealing renal tubular epithelial cells or casts correlated significantly with higher proteinuria and Scr, indicating more severe kidney damage and worse renal outcomes, thus offering potential non-invasive prognostic biomarkers (66). Additionally, presence of urinary isomorphic erythrocytes in ANCA-MPO vasculitis correlated with lower eGFR and more severe clinical presentations, suggesting utility as biomarkers for severity and progression (67). Single-cell sequencing or transcriptomics of urine sediment also shows diagnostic potential in CKD, though large prospective studies are required for validation (68, 69). Nevertheless, increased urinary tubular epithelial cells occur in acute tubular necrosis and interstitial nephritis as well, limiting specificity for renal fibrosis diagnosis.

### 2.5 Metabolites from the gut

Notably, the kidney and the gut microbiota have a complex bidirectional relationship. In CKD, dysbiosis is characterized by a decrease in beneficial bacteria (e.g., Lactobacillus, Prevotella, and Bifidobacterium) and an increase in pathogenic or opportunistic bacteria (including Proteobacteria and Enterococcus). Therefore, gut-derived metabolites can serve as biomarkers for CKD (70, 71). For example, measurement of p-cresyl sulfate (pCS) and indoxyl sulfate (IXS) in the plasma of CKD patients by LC/MS/MS showed that CKD patients had significantly higher plasma pCS and IXS, and levels were inversely correlated with eGFR. This indicates that both protein-bound solutes could serve as surrogate markers of renal function (72, 73). In a mouse model of membranous nephropathy (MN), the relative abundances of five probiotic strains (*Lactobacillus johnsonii*, *L. murinus*, *L. vaginalis*, *L. reuteri*, and *Bifidobacterium animalis*) in feces were reduced, and serum levels of indole-3-propionic acid, indole-3-aldehyde, and tryptamine were decreased (74); additionally, Cao et al. found that CKD (stages 1–5) patients exhibited gut microbiome dysbiosis, with *L. johnsonii* abundance positively correlated with eGFR, and significantly lower serum levels of indole-3-aldehyde (IAld) and 5-methoxytryptophan. Treatment of an adenine-induced CKD rat model with *L. johnsonii* and IAld improved renal injury and fibrosis, suggesting that tryptophan-derived indole metabolites may serve as predictive biomarkers in CKD (75).

Trimethylamine N-oxide (TMAO) is a dietary metabolite from choline, L-carnitine, and betaine, and the majority (>95%) of TMAO is excreted in urine (76). Multiple studies have found that when plasma TMAO levels are measured by UPLC-MS/MS or LC-MS/MS in healthy controls versus CKD patients (including DKD), CKD patients have significantly higher plasma TMAO than healthy individuals or T2DM patients. Patients on hemodialysis (HD) or peritoneal dialysis (PD) have higher TMAO levels than non-dialysis CKD patients (stages 3–5). TMAO levels correlate positively with serum creatinine, blood urea nitrogen (BUN), and uACR, and negatively with eGFR (77–81). Moreover, higher circulating TMAO levels are associated with increased mortality risk in CKD patients (82, 83). In a large 2-year cross-sectional study of healthy individuals and CKD patients, those with elevated TMAO had a higher risk of CKD, and TMAO showed moderate ability to distinguish CKD cases from non-CKD (84), therefore, TMAO has been identified as a promising biomarker; however, age, sex, body mass index (BMI), and diet may influence TMAO levels (85), and a lack of studies in CKD stages 1–3 means its sensitivity in early CKD is unclear. Large-scale studies in diverse populations are needed for validation. Additionally, plasma TMAO correlates with atherosclerosis risk (86), hence lacking specificity for renal fibrosis diagnosis.

### 2.6 Emerging technologies in noninvasive diagnosis of renal fibrosis

Advances in multi-omics approaches (including genomics, proteomics, and metabolomics) have provided new insights for the noninvasive diagnosis of renal fibrosis. Metabolomics, the study of metabolites (such as lipids, amino acids, and sugars), can reflect the metabolic state of the body at a given time, enabling early diagnosis and risk stratification (87, 88). CKD animal models induced by adenine and by unilateral ureteral obstruction (UUO), ultra-performance liquid chromatography coupled to high-definition mass spectrometry (UPLC-HDMS) revealed dysregulation of phosphatidylcholine (PC) metabolism and identified 1-methoxyphenanthrene (MP) as being associated with CKD (89), targeted analysis of blood and urine samples from healthy controls and CKD patients found that serum L-phenylalanine, L-methionine, arginine, kynurenic acid, and indoxyl sulfate, as well as urinary L-acetylcarnitine, could serve as potential biomarkers for early CKD diagnosis (90). Hong et al. (91) performed liquid chromatography-mass spectrometry (LC-MS) analysis on plasma samples from CKD stages 1–4 patients and healthy controls, and found that asymmetric dimethylarginine (ADMA), D-ornithine, L-kynurenine, kynurenic acid, 5-hydroxyindoleacetic acid, and gluconic acid were potential early biomarkers for CKD progression. Wu et al. (92) used gas chromatography-mass spectrometry (GC-MS) to analyze urinary metabolites in patients with different IgAN grades. The study found that, compared to IgAN grade 0, four volatile organic compounds (VOCs) were significantly elevated in grade 1; and compared to grade 1, two additional VOCs were upregulated in grades ≥2. These results suggest that urinary VOCs might serve as noninvasive biomarkers reflecting the dynamic progression of CKD via fibrotic changes. Additionally, Peters et al. (93) conducted proteomic analysis of urine samples from IgAN patients and identified a proteomic classifier called “IgAN237” that has predictive value for disease progression. This classifier provides a promising biomarker for risk stratification and longitudinal monitoring in IgAN.

Furthermore, Doke et al. (32) applied single-cell RNA sequencing (scRNA-seq) to kidney tissues from CKD patients and healthy controls, finding increased basophil infiltration in fibrotic kidneys and showing that IL-6, IL-18, and IL-33 expression in the kidney correlated with CKD severity. At the same time, comparative serum proteomic analysis showed that levels of heat shock protein 90β family member 2 (HSP90B2) and α1-antitrypsin (AAT) were elevated in CKD patients compared to healthy individuals, and these levels correlated positively with known clinical markers, suggesting that they could serve as novel CKD biomarkers (94).

Machine learning (ML) has become a key artificial intelligence tool in microbiome research. Metabolomic analysis combined with ML can reveal metabolic differences between CKD patients and healthy controls and validate those differences, providing new possibilities for CKD management (95). Chen et al. (96) performed metabolomic profiling on serum samples from 703 CKD stages 1–5 patients. They found that 5-methoxytryptophan (5-MTP), adrenosterol succinate, tiglylcarnitine, and taurine were negatively correlated with CKD progression, whereas acetylcarnitine was positively correlated. Validation with ML showed that this panel of five metabolites could effectively distinguish CKD stages 1–5 patients, indicating that these metabolites could serve as early CKD biomarkers. In a retrospective cross-sectional study using LC-MS/MS-based metabolomics, plasma levels of tryptophan (Trp) derivatives were quantified in healthy controls and CKD patients (including IgAN). The study found that plasma melatonin had >95% accuracy in diagnosing early-stage CKD (stages I–II); furthermore, indole-3-lactic acid showed excellent ability to distinguish IgAN among CKD patients (97). Wu et al. (98) performed full-length 16S rRNA gene sequencing on fecal samples from healthy controls, T2DM patients, CKD patients, and diabetic kidney disease (DKD) patients, combined with ML analysis. They found that levels of L-valine, L-leucine, and L-isoleucine, and their precursor L-glutamate, were significantly increased in DM and DKD patients, suggesting these may serve as potential diagnostic biomarkers for DKD. Hirakawa et al. (99) integrated untargeted metabolomic profiles of plasma and urine from DKD patients with ML and found that systolic blood pressure, urine albumin-to-creatinine ratio (uACR), and certain metabolites (such as urinary N-methylproline, NMP) could serve as candidate biomarkers; however, these metabolites still require external validation.

### 2.7 Combined use of biomarkers

In CKD stages 2–5, simultaneous measurement of an inflammatory marker (IL-6), lipid markers, and kidney injury indices revealed that IL-6, BUN, and hemoglobin (Hb) levels differed significantly across stages, and these markers were risk factors for disease progression. This suggests that combining serum biomarkers can enable dynamic monitoring of CKD progression, aid in risk stratification, and guide early therapeutic intervention (100). Using a combination of serum and urinary biomarkers can improve diagnostic accuracy, provide a more comprehensive overview of renal health, and allow better risk stratification and personalized treatment planning.

### 2.8 Other biomarkers

Studies have shown that angiopoietin-like protein 4 (ANGPTL4) expression is significantly upregulated in CKD rats and patients, suggesting ANGPTL4 may be a novel noninvasive marker of renal fibrosis (101, 102). In one cohort study, plasma TNFR-1, YKL-40, and KIM-1 were associated with the risk of requiring kidney failure replacement therapy (KFRT) in diabetic patients (103).

Additionally, other molecules—including CDH11, SERPINF1 (also known as PEDF), SMOC2, HNF4A, NELL1; soluble lymphatic endothelial hyaluronan receptor 1 (sLYVE1); and markers such as CD44, nicotinamide N-methyltransferase (NNMT), and galactosylceramidase A-9—have shown significant associations with interstitial fibrosis/tubular atrophy (IFTA). These molecules have been identified as biomarkers of renal fibrosis (104–109). However, more and larger clinical sample data are needed to support these findings. Creatinine measured in fingernails correlates with serum creatinine in CKD patients (110). Retinal fundus examination (111) and retinal imaging combined with deep learning (112, 113) have been applied to CKD and T2DM detection and risk stratification. However, these methods also require larger clinical samples and external validation.

## 3 Imaging techniques

### 3.1 Ultrasound

Conventional renal ultrasound is primarily used to evaluate nephrolithiasis and mass lesions. However, due to factors such as anatomical position, respiratory motion, and limited resolution, traditional ultrasound is not sensitive for detecting renal fibrosis. With advances in ultrasound imaging, techniques like elastography and photoacoustic imaging have emerged, providing new methods for diagnosing renal fibrosis.

#### 3.1.1 Ultrasound elastography

Ultrasound elastography (USE) assesses renal fibrosis by measuring changes in tissue elasticity. There are two main approaches: (1) strain elastography (SE), which analyzes tissue deformation (strain) under external pressure; and (2) shear wave elastography (SWE), which evaluates tissue stiffness by measuring the velocity of ultrasound-induced shear waves (shear wave velocity, SWV) (114). The measurement of SWE between CKD patients and health showed that the cortical hardness of CKD patients was significantly increased, which was positively correlated with serum urea/creatinine levels and negatively correlated with GFR (115). A prospective study demonstrated that SWE-based Young’s modulus measurements had high sensitivity and specificity for diagnosing interstitial fibrosis in IgAN patients (116). In 162 CKD patients who underwent 2D-SWE and kidney biopsy, the use of machine learning algorithms (XGBoost and MLP models) enabled differentiation between severe and mild renal fibrosis (117, 118). Logistic regression analysis showed that combining eGFR with SWE values improved diagnostic accuracy for mild, moderate, and severe fibrosis in CKD patients (118). Similarly, Zhu et al. (119) found that combining SWE with a support vector machine (SVM) model enhanced the ultrasound diagnosis of different grades of tubulointerstitial fibrosis in CKD patients.

#### 3.1.2 Photoacoustic imaging

Photoacoustic imaging (PAI) is a noninvasive technique that combines ultrasound and laser light, using optical absorption to generate image contrast. Because different tissue components have distinct optical absorption properties, PAI can quantify renal collagen content and reflect the progression of fibrosis (120). Photoacoustic collagen imaging has been used to rapidly and noninvasively quantify renal fibrosis burden in isolated mouse and pig kidneys, as well as cortical fibrosis in ex vivo human kidneys (121). Multiple studies in animal models of renal fibrosis have shown that the use of nanoparticles in conjunction with PAI provides potential for longitudinal staging in clinical fibrosis assessment (122, 123). Although PAI is very promising for quantifying collagen content and evaluating fibrosis, its application in human renal fibrosis has been seldom studied due to issues such as suboptimal biocompatibility, limited penetration depth, and low resolution.

#### 3.1.3 Contrast-enhanced ultrasound and super-resolution ultrasound

Microbubble contrast agents, not excreted through kidneys, can safely be used in renal impairment. Contrast-enhanced ultrasound (CEUS) monitors arterial microbubble arrival, cortical enhancement, and subsequent medullary filling, while super-resolution ultrasound (SRUS) reveals microvasculature with high-resolution imaging of microbubble trajectories (124–126). Studies using CEUS in CKD found significantly reduced cortical microperfusion correlating with eGFR, although differences among CKD subgroups were not observed (127–129). SRUS precisely quantified progressive pathological changes, including reduced renal size, cortical thickness, and altered microvascular structure in mouse models (130). SRUS also identified decreased vascular density in CKD patients and accurately depicted renal transplant microvasculature (131–133). Zhao et al. (134, 135) developed a hybrid PA/SRUS imaging method for simultaneous monitoring of renal oxygenation and hemodynamics. Both CEUS and SRUS have potential as diagnostic tools for progressive kidney disease monitoring but face clinical limitations such as motion artifacts and imaging speed, requiring further validation.

#### 3.1.4 Superb microvascular imaging

Superb microvascular imaging (SMI), a novel vascular imaging technique without contrast agents, detects slow blood flow in small vessels with high frame rate, reduced motion artifacts, and high resolution. CKD stage 2–5 patients (including T2DM and hypertension) exhibited significantly lower SMI vascular indices compared to controls, correlating moderately with SCr and eGFR and aligning with histological changes and CKD stages (136). Thus, SMI can assess morphological renal changes and stage differentiation in CKD patients.

#### 3.1.5 Artificial intelligence and ultrasound

Artificial intelligence (AI) leverages non-invasive data to predict pathological outcomes. Chang et al. (137) integrated ultrasound images and biomarkers (creatinine, age, gender, and proteinuria) of CKD patients into ML models, accurately predicting IFTA and providing a powerful non-invasive early CKD assessment tool. DL models based on SMI were superior to ultrasound radiomics and CDUS models in determining IF severity in CKD (138). Qin et al. (139) demonstrated excellent accuracy of a DL model combining grayscale US, SMI, and SE for early prediction of chronic renal fibrosis.

Ultrasound, widely utilized for non-invasive CKD assessment, has yet to clinically integrate CEUS and SMI fully due to renal position, motion artifacts, and cost. Nevertheless, SMI and ultrasound combined with AI exhibit substantial potential for renal fibrosis diagnosis.

### 3.2 Magnetic resonance imaging

Magnetic resonance imaging (MRI) is a powerful tool for assessing the structure and function of both kidneys. Renal fibrosis alters water diffusion patterns, oxygenation, perfusion, and tissue stiffness. Accordingly, MRI can evaluate renal fibrosis through various imaging techniques. Functional MRI modalities such as diffusion-weighted imaging (DWI), diffusion tensor imaging (DTI), intravoxel incoherent motion (IVIM) imaging, diffusion kurtosis imaging (DKI), blood oxygen level-dependent MRI (BOLD-MRI), and arterial spin labeling (ASL) have garnered increasing attention in CKD research. These methods allow dynamic monitoring of microstructural and functional changes in the kidneys and can quantitatively assess diffusion, fibrosis, and oxygenation without the need for exogenous contrast agents (140).

#### 3.2.1 Diffusion MRI

In fibrotic kidneys, ECM deposition and tubular atrophy limit water diffusion, so diffusion MRI based on the pattern of water molecule diffusion in renal tissue can reflect the structure and spatial organization of the kidney.

##### 3.2.1.1 DWI

DWI detects the random diffusional motion of water molecules and quantifies this process via the apparent diffusion coefficient (ADC). Renal fibrosis restricts water molecule diffusion, leading to a decrease in ADC values in the kidney (120, 141). Some studies have noted that in animal models of diabetic nephropathy and in patients with renal artery stenosis, renal ADC values are reduced and show a negative correlation with the degree of interstitial fibrosis (142–144).

##### 3.2.1.2 DTI

DTI is an extension of DWI that evaluates the directional movement of water molecules and quantifies it using fractional anisotropy (FA). Renal fibrosis can lead to interstitial fibrosis, glomerulosclerosis, and inflammatory infiltrates, all of which can affect FA values. Studies in transplant kidneys have shown that medullary FA and ADC are significantly reduced, with both FA and ADC correlating positively with eGFR and negatively with fibrosis extent (145).

Moreover, the AUC of DTI was greater than that of ASL in accurately identifying allograft fibrosis (146). Other research has shown that in CKD patients of various etiologies (such as lupus nephritis) versus healthy controls, cortical FA values are significantly higher while ADC values are significantly lower in the patient group, indicating that DTI is a valuable noninvasive tool for assessing renal dysfunction and fibrosis (147). In addition, in CKD patients, cortical FA and ADC may help distinguish DN patients from healthy individuals and assess fibrosis severity (148). Therefore, DTI is a promising noninvasive method for evaluating renal dysfunction and fibrosis.

##### 3.2.1.3 IVIM

IVIM-DWI can simultaneously assess the diffusion coefficient (D) of water molecules, the perfusion-related pseudo-diffusion coefficient (D*), and the perfusion fraction (f) (149). Mao et al. (149) compared IVIM parameters (D, D*, f) between CKD patients and healthy volunteers and found that all IVIM parameters were significantly lower in CKD patients than in controls. Additionally, renal parenchymal IVIM parameters were negatively correlated with fibrosis scores. In patients with DN, IgAN, and non-diabetic renal disease (NDRD), MRI parameters were significantly associated with interstitial fibrosis/tubular atrophy scores, suggesting that IVIM-DWI can aid in noninvasively evaluating renal function, fibrosis extent, and prognostic risk in DN and IgAN (150, 151), however, in a study by Ichikawa of CKD patients with varying disease severity, cortical D* values were lower in moderate-to-severe CKD than in mild CKD, whereas cortical f did not differ significantly between groups (152). Thus, IVIM shows promise for noninvasive assessment, but its utility in grading fibrosis severity requires further validation.

##### 3.2.1.4 DKI

DKI is an extension of DTI that quantifies the non-Gaussian distribution of water molecule diffusion in tissue; it provides metrics such as the kurtosis (K) index, an apparent kurtosis coefficient (K), and a diffusion coefficient (D) analogous to ADC. Liu et al. (153) used DKI to evaluate renal fibrosis in IgAN and CKD patients and found that the kurtosis (K) value was significantly correlated with fibrosis scores, and it performed better in identifying severe IF/TA. This suggests that DKI can serve as a noninvasive method for detecting renal fibrosis in IgAN and CKD patients. In CKD patients, a histogram analysis of DKI-derived D and K values showed that the 90th percentile of cortical K and D had the strongest correlations with fibrosis scores. This histogram analysis demonstrated feasibility in assessing changes in renal function and fibrosis in CKD patients (154). Furthermore, in patients with primary kidney diseases, hypertension, or diabetes, DKI metrics such as mean diffusivity (MD) and interstitial fibrosis are negatively correlated, while axial kurtosis (K_a) is positively correlated with interstitial fibrosis. This suggests that DKI has potential applications in monitoring renal interstitial fibrosis (155).

#### 3.2.2 BOLD-MRI

BOLD imaging uses the transverse relaxation time (T2* = 1/R2*) to assess tissue oxygenation. In fibrotic kidneys, deoxyhemoglobin levels are elevated and T2* relaxation time is shortened (156). In CKD patients, including those with non-diabetic renal disease (NDRD), the rate of eGFR decline is significantly associated with both cortical and medullary T2* values, suggesting that BOLD-MRI may provide a noninvasive method to assess the severity of renal injury (157). In CKD patients, including those with non-diabetic renal disease (NDRD), the rate of eGFR decline is significantly associated with both cortical and medullary T2* values, suggesting that BOLD-MRI may provide a noninvasive method to assess the severity of renal injury (158–161). However, in DN patients, renal T2* did not correlate with eGFR (162). Researchers have shown that factors such as the type and severity of kidney disease, plasma sodium concentration, fluid intake, hematocrit, microvascular density, and renal blood volume can influence T2* values, indicating that the effectiveness of BOLD-MRI in assessing renal fibrosis remains a subject of debate (163, 164).

#### 3.2.3 ASL MRI

Arterial spin labeling (ASL) is a quantitative MRI technique based on tissue perfusion; it uses magnetically labeled arterial blood water as an endogenous tracer to measure renal perfusion (reported in mL/100 g/min) (165). In an allograft transplant model, renal perfusion was decreased and was inversely correlated with fibronectin expression. ASL-MRI studies comparing transplant recipients to healthy controls found that peritubular capillary density was significantly reduced in transplanted kidneys, and that cortical renal blood flow (RBF) decreased with increasing fibrosis, with a moderate negative correlation to Banff fibrosis scores (166, 167). Additionally, in CKD patients (including those with DN and IgAN), RBF declines as interstitial fibrosis worsens. Morra-Gutierrez reported a significant difference in cortical perfusion when IF exceeded 30%, while Mao et al. (168) reported that the ROC AUCs for ASL-derived RBF were 0.93 and 0.90 in distinguishing ≤25% vs. >25% IF and ≤50% vs. >50% IF, respectively (169, 170). Therefore, ASL can serve as a predictor of DKD progression and fibrosis; however, due to low signal-to-noise ratio and resolution limitations, larger longitudinal studies are needed to evaluate its potential for CKD and fibrosis stratification. Therefore, ASL can serve as a predictor of DKD progression and fibrosis; however, due to low signal-to-noise ratio and resolution limitations, larger longitudinal studies are needed to evaluate its potential for CKD and fibrosis stratification.

#### 3.2.4 Magnetic resonance elastography

Similar to SWE, magnetic resonance elastography (MRE) can be used to assess tissue stiffness. In an adenine-induced rat model of renal fibrosis, MRE showed significantly increased shear wave speed (SWS) in fibrotic kidneys, and this SWS was positively correlated with the collagen area fraction (CAF) (171). In kidney transplant patients with biopsy-confirmed fibrosis, renal stiffness measured by MRE correlated positively with Banff fibrosis scores (172). However, a study by Chauveau et al. (173) found no significant correlation between MRE-derived stiffness and Banff fibrosis scores, nor an inverse correlation between stiffness and cortical glomerulosclerosis rate, suggesting that reduced renal blood flow might explain these discrepancies. Similarly, mixed results have been observed in patients with DN, IgAN, and LN. Brown reported that renal stiffness gradually decreased as DN progressed, while renal blood flow measured by ASL also declined significantly; the latter showed a strong positive correlation with both eGFR and MRE-derived shear stiffness (170). Furthermore, healthy individuals exhibited increased renal stiffness after water loading, indicating that increased renal perfusion pressure can raise tissue stiffness (174). Thus, while MRE does reflect the presence of renal fibrosis, factors such as renal perfusion contribute to variability in measurements.

#### 3.2.5 Other novel MR techniques

In patients with chronic glomerulonephritis (CGN), native T1 relaxation times are significantly elevated, closely correlating with CKD stage. ROC analysis showed that the optimal T1 threshold for predicting renal fibrosis was 1,695 ms (specificity 0.778, sensitivity 0.625). Therefore, native T1 mapping can be an effective, noninvasive method for detecting renal fibrosis in CGN patients (175). MPI is an innovative functional imaging modality exploiting SPION nonlinear responses to achieve three-dimensional lesion localization. Its high sensitivity, temporal resolution, and quantitative measurement capabilities make it highly suitable for preclinical molecular imaging applications (176).

Additionally, as noted above, fibroblast activation protein (FAP) can serve as a fibrosis biomarker. Combining MRI with a FAP-targeted fluorescent probe enabled highly sensitive imaging of fibrotic kidneys in a UUO mouse model, demonstrating potential for early RF diagnosis and guidance of FAP-targeted therapy (177). Using amide proton transfer-weighted MRI (APTw) in bilateral renal ischemia-reperfusion (IRI) and UUO models, researchers found that cortical APT (cAPT) and medullary APT (mAPT) values were positively correlated with the extent of renal fibrosis; in early-stage fibrosis, APT values had better diagnostic performance than ADC values (178). Gd-OA, a probe targeting collagen side chains designed by Chen et al. (179), and newly synthesized gadolinium oxide nanoparticles (Gd₂O₃ NPs) by Ashouri et al. (180), have been shown in combination with MRI to be important tools for detecting and staging renal fibrosis in animal models. However, further research is needed to determine their clinical applicability.

In summary, diffusion-based MRI techniques—including DWI, DTI, IVIM, and DKI—have shown potential value for noninvasive diagnosis of renal fibrosis. Studies have found significant differences in parameters such as ADC, D, D*, f_p, mean kurtosis (MK), and MD between CKD stages 1–2 and 3–5 in patients and healthy volunteers. Moreover, renal MD, D, and medullary FA are negatively correlated with injury scores. Thus, IVIM appears to have higher diagnostic value than DWI in CKD patients. However, another study noted that DKI and medullary DTI outperformed DWI and IVIM in evaluating the severity of renal pathology and dysfunction in CKD (181, 182). Additionally, in IgAN patients and healthy volunteers, multiple MRI parameters (cortical and medullary T2^*^, ADC, D, D^*^, and f) decreased with declining eGFR. Except for cortical and medullary D^*^, all MRI parameters were significantly correlated with interstitial fibrosis scores, with cortical D^*^ showing the strongest correlation. Therefore, IVIM-DWI and BOLD-MRI can aid in further assessing renal function, Oxford classification lesions, and prognostic risk in IgAN patients (150).

### 3.3 Computed tomography and positron emission tomography/computed tomography

#### 3.3.1 CT

Because iodinated contrast agents may worsen renal insufficiency, contrast-enhanced computed tomography (CT) is rarely used in the clinical diagnosis of kidney diseases (183). Radiomics employs advanced algorithms to convert standard medical images into high-dimensional data arrays, capturing subtle renal structural changes. CT combined with ML demonstrated higher diagnostic accuracy (AUC) in differentiating CKD stages 1–3 from healthy controls compared to radiologists (184). A CNN model developed by Chantaduly et al. (185) differentiated mild/moderate from severe renal fibrosis similarly to renal biopsy. Ren et al. (186) reported superior performance of combined radiomics models in predicting IF grading (mild–moderate vs. severe).

#### 3.3.2 PET/CT

PET/CT offers high specificity and sensitivity by using molecular probes that target specific biological processes or molecules to quantitatively assess radiotracer accumulation in fibrotic tissue, thereby elucidating disease mechanisms (187). In normal organs, FAP is barely detectable, but it is significantly upregulated in areas of tissue remodeling, including renal and pulmonary fibrosis (188). Huang et al. (189) employed PET/CT with FAP-targeted tracers ([^18^F]FAPI-42 and [^18^F]AlF-NOTA-FAPI) to image kidneys on day 2 after acute kidney injury (AKI). They found that AKI was associated with renal fibrosis by day 14, and that FAP-specific PET/CT imaging was able to dynamically observe the maladaptive repair process after AKI and predict the development of renal fibrosis. The radiolabeled FAP inhibitor [^68^Ga]Ga-FAPI-04 has been demonstrated as an imaging tracer for PET/CT. Our team was the first to perform [^68^Ga]Ga-FAPI-04 PET/CT in an adenine-induced CKD model, reporting increased renal FAPI uptake that rose over time and correlated with the extent of renal fibrosis (190). We subsequently applied this imaging in CKD patients undergoing biopsy, and the results showed that nearly all patients with renal fibrosis exhibited tracer uptake, which increased with fibrosis severity, indicating that [^68^Ga]Ga-FAPI-04 PET/CT can sensitively detect renal fibrosis at early stages (191). Additionally, a peritoneal fibrosis (PF) rat model showed significantly increased ^68^Ga tracer uptake compared to controls (192). PET/CT imaging with [^68^Ga]Ga-FAPI-04 was also performed in LN patients and healthy individuals. The study found that renal uptake of ^68^Ga-FAPI-04 was positively correlated with disease progression, serum creatinine, chronicity index, and the degree of tubulointerstitial fibrosis. LN patients had significantly higher renal ^68^Ga-FAPI-04 uptake than healthy controls, suggesting that ^68^Ga-FAPI-04 PET/CT can be used for noninvasive assessment of tubulointerstitial fibrosis in active lupus nephritis (193). Conen et al. (194) conducted a retrospective analysis of patients who underwent [^68^Ga]Ga-FAPI PET/CT and reported that renal parenchymal FAPI uptake was significantly inversely correlated with eGFR, indicating that [^68^Ga]Ga-FAPI has potential as a noninvasive tool for CKD staging and quantitative assessment. Furthermore, in a trial of 14 patients with histologically confirmed Erdheim–Chester disease (ECD), ^68^Ga-FAPI PET/CT outperformed ^18^F-FDG PET/CT, including showing enhanced image contrast and higher lesion SUV max across multiple organs (kidneys, heart, lungs) (195). Wang et al. (196–198) reported the use of ^18^F-AlF-NOTA-FAPI-04 PET/CT in a patient with multiple myeloma and renal interstitial fibrosis. The scan showed markedly increased FAPI uptake in both kidneys. In a comparison with healthy individuals, patients with IgAN, MN, or DN had significantly higher renal uptake on ^18^F-AlF-NOTA-FAPI-04 PET/CT. Moreover, renal SUV_max correlated positively with interstitial fibrosis, tubular atrophy, and tubulointerstitial inflammation scores on biopsy, suggesting that ^18^F-AlF-NOTA-FAPI-04 PET/CT may be valuable for noninvasive evaluation of renal interstitial fibrosis and for monitoring disease progression. The authors proposed that ^18^F-AlF-NOTA-FAPI-04 could serve as an alternative to [^68^Ga]Ga-FAPI. If FAPI-targeted imaging is validated in larger dedicated studies and proven to be specific for fibrosis, it may represent the first direct noninvasive imaging approach for renal fibrosis (199).

Cardiorenal syndrome is defined as a pathophysiological disorder including both heart and kidneys (200). Brown has suggested that ^13^N-ammonia PET/CT can simultaneously evaluate myocardial and renal perfusion. In his study, resting PET-measured renal blood flow was strongly negatively correlated with histological interstitial fibrosis, opening a potential new avenue to investigate therapies that confer overlapping benefits to the heart and kidneys (201). In summary, PET/CT provides a comprehensive assessment of renal structure and function, effectively monitors CKD and renal fibrosis progression, and offers opportunities for early detection, accurate diagnosis, and personalized therapeutic strategies.

### 3.4 Radiomics

With technological advancements, radiomics holds promise for providing more reliable and accurate information on diagnosis, treatment, and prognosis. Lu et al. (202) developed a multimodal molecular imaging system integrating PET, SPECT, FMI, and CT to obtain comprehensive small animal imaging data. The kidney imaging project (KIP) initiated by Zhou et al. (203) aims to advance precision nephrology through multimodal, multi-scale renal imaging atlases.

## 4 Discussion and conclusions

**Figure 3:**
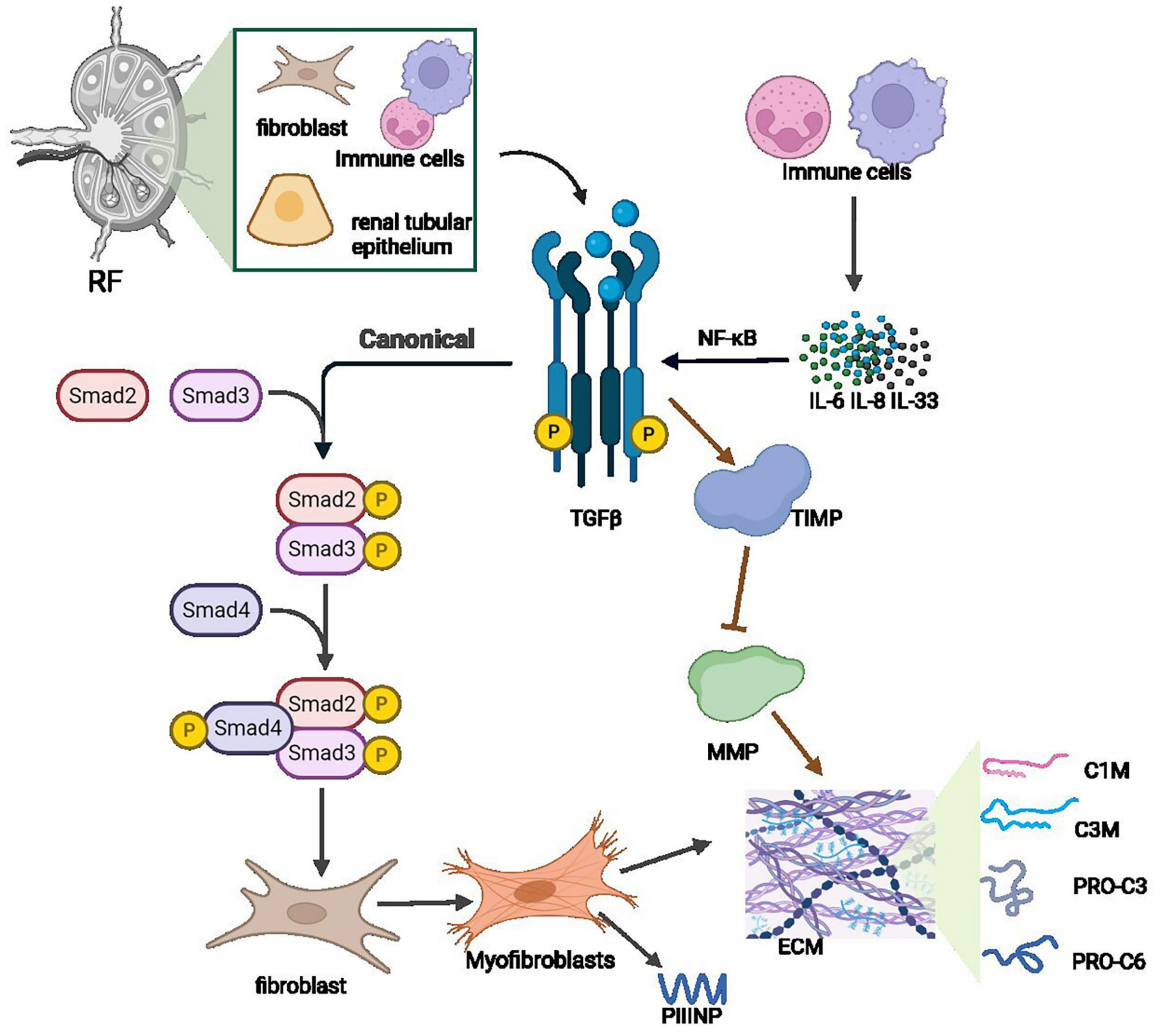
Key hematological biomarkers for noninvasive diagnosis of renal fibrosis.

In conclusion, despite renal biopsy remaining the diagnostic gold standard for renal fibrosis, it is limited by invasiveness, bleeding risks, and sampling inadequacies. Various serum and urinary biomarkers are crucial for early diagnosis, dynamic monitoring, and therapeutic evaluation (Table 2). Biomarkers like TGFβ1 reflect fibrosis and inflammation activation; pro-fibrotic cytokines (e.g., IL-6) correlate with renal dysfunction and fibrosis progression. Urinary biomarkers (KIM-1, NGAL) directly reflect local pathology and ongoing tubular injury; collagen metabolites, exosome-derived miRNAs (miR-21, miR-29), and TIMPs relate to abnormal ECM deposition (Figure 3). However, due to limited assay standardization (e.g., ELISA) and insufficient specificity, combined use of biomarkers with multi-omics (proteomics, metabolomics) and ML has gained attention. Clinical translation remains challenged by assay standardization and pathology correlations. Integration of biomarkers with radiomics and AI may enhance fibrosis assessment precision in the future.

**Table 2:** Blood or urine markers for diagnosing kidney fibrosis.

Classification	Biomarker	Type of disease (person)	Sample type	Advantage	Disadvantage
ECM	PRO-C3_,_ PRO-C6 and C1M, C3M	AAV kidney involvement (8) LN (9), IgAN (10), T2DM (11)	Blood, urine	Related to collagen synthesis/degradation	Interfered with by fibrosis of other organs
	MMPs	T2DM (15) Hypertension (16) DKD and IgAN (17, 18)	Blood, urine	Hypertension and T2DM are early indicators of CKD progression Related to RF	Thresholds are not uniform
Biomarkers associated with inflammation activation	TGFβ1	IgAN (22), DKD (25), LN (12)	Blood, urine	Direct involvement in the fibrosis process correlated with eGFR and TIF	Extensive involvement in fibrosis of other organs Not specific
	MCP-1	DKD (27, 28), CKD with hypertension (29), LN (30)	Blood, urine	Increased significantly at the initiation stage of fibrosis	Interfered by urinary tract infection
	ILs	CKD with hypertension or T2DM (IL-6, IL-18 and IL-33) (32), DKD (IL-6) (33) Kidney transplant (IL-8 mRNA) (34), IgAN (IL-7) (35) DKD (IL-22)	Blood, urine	Increased in CKD Related to eGFR and degree of fibrosis	Does not increase the predicted risk of disease progression
Tubular injury markers	KIM-1	ANCA-GN (39)	Blood, urine	Indicates tubular injury and CKD severity	Does not increase the predicted risk of disease progression Factors such as infection and ischemia interfere
	NGAL	CTIN_,_ MN (42), T2DM (36)	Blood, urine		
	NAG	DKD (44)	Blood, urine		
	DKK-3	CKD (48) kidney transplant (49) PD (51)	Urine	Increased in CKD Related to eGFR and degree of fibrosis	Lack of large-scale queue validation
	VCAM1	MLN, PLN (53, 54) T2DM, DKD (55) LN (56)	Blood	Associated with the degree of renal interstitial inflammation and fibrosis score	Elevated in AS and infection Low specific
EVs	mRNA	DKD_,_ FSGS_,_ IgAN_,_ MN (CD2AP mRNA) (59) Kidney transplant (VTN) (60)	Urine	Reflects the physiological state of the source cell	Lack of consensus on standardization of urine collection, processing and storage and on uEV separation and downstream analysis
	miRNA	Peritoneal dialysis (miR-21) (62) IgAN (miR-451a) (63)	Urine		
Urine sediment and urinary exfoliated cell	Renal tubular epithelial cells or casts urinary isomorphic erythrocytes	DKD (66) ANCA-MPO vasculitis (67)	Urine	Directly reflects the core link of tubular injury	Impossible to distinguish between AKI and CKD
Metabolites from the gut	Pcs, IXS, indole-3-propionic acid, indole-3-aldehyde, and tryptamine	CKD (72) MN (74)	Blood	Associated with the fibrosis score	Interfered by liver function and protein intake
	TMAO	T2DM, CKD (77–84)		Positively correlated with Scr, BUN and UACR, and negatively correlated with eGFR	Affected by age, sex, BMI, and diet Correlated with AS Low specific

Biomarkers: PRO-C3, procollagen III N-terminal propeptide; PRO-C6, procollagen VI C-terminal propeptide; C1M, collagen type I degradation marker; C3M, collagen type III degradation marker; MMPs, matrix metalloproteinases; TGFβ1, transforming growth factor beta-1; MCP-1, monocyte chemoattractant protein-1; ILs, interleukins; DKK-3, Dickkopf-related protein 3; VCAM1, vascular cell adhesion molecule 1; TMAO, trimethylamine N-oxide; KIM-1, kidney injury molecule-1; NGAL, neutrophil gelatinase-associated lipocalin; NAG, N-acetyl-β-D-glucosaminidase; VTN, von Willebrand factor-associated nephropathy. Diseases and pathology: LN, lupus nephritis; IgAN, immunoglobulin A nephropathy; T2DM, type 2 diabetes mellitus; ANCA-GN, ANCA-associated glomerulonephritis; D-CTIN, drug-induced chronic tubulointerstitial nephritis; MN, membranous nephropathy; PD, peritoneal dialysis; MLN, membranous lupus nephritis; PLN, primary lipoprotein glomerulopathy; FSGS, focal segmental glomerulosclerosis; ANCA-MPO, vasculitis, myeloperoxidase-ANCA vasculitis; AAV, ANCA-associated vasculitis. Others: TIF, tubulointerstitial fibrosis; AS, atherosclerosis; BMI, body mass index.

Advancements in ultrasound technologies (SRUS, SMI) are transitioning renal fibrosis diagnostics from macroscopic to microcirculatory, molecular, and functional multidimensional assessments. Despite limitations, multimodal imaging with AI may eventually replace biopsies. Imaging methods (DWI, DTI, IVIM) show inconsistent correlations with fibrosis. Enhanced CT and MRI pose risks; radiomics and AI may improve diagnostic accuracy but need further clinical validation. PET/CT combined with FAPI shows high specificity and sensitivity, offering promising quantitative diagnostic potential for renal fibrosis (Table 3).

**Table 3:** Characteristics of noninvasive renal fibrosis imaging techniques.

Detection	Method	Advantage	Disadvantage	Clinical application	Diagnostic value
Tissue stiffness	USE	Non-invasive, real-time imaging, low cost, no radiation	Dependent on the operator’s experience, sensitive to obesity or gas interference, with limited depth	Initial screening and dynamic monitoring of fibrosis p regression	Dependent on the operator’s experience, sensitive to obesity or gas interference, with limited depth
	MRE	Quantifiable, high accuracy, unaffected by operator influence	High equipment requirements and low adoption rate	During the research phase, potential non-invasive evaluation tools	The reflection of kidney fibrosis by MRE remains controversial due to factors such as renal perfusion
Diffusion of water molecules	DWI	Noninvasive, no contrast agent	It is susceptible to respiratory motion artifacts and has low resolution	Auxiliary diagnosis of fibrosis and differentiation of other kidney diseases (such as inflammation)	It needs to be combined with other sequences, and its sensitivity and specificity need to be improved

	DTI	Assess structural disorder caused by fibrosis	The scanning time is long, the motion is sensitive, and the clinical application is limited	Conducted to assess early fibrosis in DN or hypertensive kidney damage	Lack of standardized parameter threshold, the research is mostly in the experimental stage
IVIM	Distinguish between perfusion and diffusion	Post-processing is complex	Early microcirculation assessment	The severity of renal fibrosis needs to be further verified

DKI	Sensitive detection of heterogeneity	High equipment requirements	Research evaluates complex fibrosis	Potential applications in renal interstitial fibrosis
The collagen content	PAI	Molecular targeting potential	Poor biocompatibility, limited detection depth and low resolution	Experimental studies or targeted therapy evaluation	Few studies on its application in clinic
Tissue oxygenation	BOLD MRI	Noninvasive assessment of hypoxia	Specificity is low, factors such as plasma sodium concentration, water intake, renal blood volume were affected	Monitor chronic hypoxia injury	Indirect indicator
Tissue perfusion	ASL-MRI	No contrast agent is required and it can be repeated	The signal-to-noise ratio is low, the motion is sensitive, and the technology has not been fully standardized	Monitor ischemic changes associated with fibrosis	Indirectly reflects the degree of fibrosis (related to renal function)
Radioactive concentration accumulated in fibrotic tissues	PET/CT	Functional imaging, high specificity and sensitivity reflecting pathophysiological changes	Radiation exposure, low resolution and high cost	Research areas (such as the development of targeted fibrosis molecular probes)	At present, it is mainly used for scientific research, and its clinical routine application is limited

In conclusion, despite significant advancements and the demonstrated potential of serum and urinary biomarkers and imaging techniques such as ultrasound, PET/CT, and MRI, much work remains to be done to clarify biomarker mechanisms, enhance imaging diagnostic thresholds, and expand the clinical translation of these methods. Future efforts should focus on integrating novel biomarkers and imaging modalities with multi-omics and artificial intelligence approaches to overcome the current “bench-to-bedside” translation bottleneck, enabling earlier diagnosis and treatment of renal fibrosis.
